# P69. Targeting naturally presented, leukemia-derived HLA ligands with TCR-transgenic T cells for the treatment of therapy refractory leukemias

**DOI:** 10.1186/2051-1426-2-S2-P43

**Published:** 2014-03-12

**Authors:** K Richard, S Schober, M Rami, S Mall, J Merl, J Slotta-Huspenina, S Stevanovic, DH Busch, C Peschel, AM Krackhardt

**Affiliations:** 1 Klinikum rechts der Isar - Technische Universität München, III. Medizinische Klinik, München, Germany; 2 Helmholtz Zentrum München (GmbH), Research Unit Protein Science, Neuherberg, Germany; 3 Klinikum Rechts der Isar - Technische Universität München, Institut für Allgemeine Pathologie und Pathologische Anatomie, Munich, Germany; 4 University of Tübingen, Department of Immunology, Tübingen, Germany; 5 Technische Universität München, Institut für Medizinische Mikrobiologie Immunologie und Hygiene, Munich, Germany

## Background

T cells have proven to be effective for the treatment of leukaemias in form of donor lymphocyte infusions (DLI). However, those DLI are of unknown specificity and often associated with graft versus host disease. New effector tools in form of T cell receptor (TCR)-transgenic T cells with specificity for leukaemia-derived human leukocyte antigen (HLA) ligands are highly promising tools for the treatment of life-threatening, therapy refractory leukaemias.

## Material and methods

In order to identify leukaemia-derived HLA ligands, we performed the immunopeptidomic approach on seven samples from patients with myeloproliferative neoplasias (MPN). HLA ligands derived from genes with expression restricted to the hematopoeitc system were identified by database searches and literature research and validated by quantitative PCR (qPCR) and immunohistochemistry (IHC). For isolation of peptide-specific TCR, naive T cells of healthy blood donors were primed with single HLA-mismatched dendritic cells, pulsed with synthetic counterparts of selected HLA ligands. Peptide specific T cells were isolated using HLA multimers and cloned by limiting dilution. TCR were isolated out of peptide-specific T cell clones and characterised for their *in vitro* and *in vivo* leukaemia reactivity as well as their *in vitro* on- and off-target reactivity.

## Results

Using the immunopeptidomic approach on seven MPN samples, we were able to identify 4386 unique HLA ligands. Nineteen of those ligands are derived from seven genes with restricted expression to the hematopoietic system and are presented on six different HLA molecules. We exemplarily selected the antigen myeloperoxidase (MPO) (five identified ligands) and confirmed restricted expression to myeloid cells. A TCR (named TCR2.5D6) with high specificity for the HLA-B*07:02 restricted ligand MPO_5_ could be isolated. TCR2.5D6-transgenic T cells show *in vitro* leukaemia reactivity including colony forming leukaemic progenitor cells. Strikingly, anti-leukaemic reactivity could be observed in a murine model of human acute myeloid leukaemia with HLA-B*07:02-transgenic NB4 cells, resulting in significantly prolonged survival and reduced bone marrow infiltration by leukaemic cells. However, strong immune pressure led to the development of tumours that lost transgene expression in the TCR treated group. Extensive experiments regarding safety of the TCR2.5D6 revealed lack of reactivity against MPO^-^ cells including healthy hematopoietic stem cells. Experiments with alanine variants of the MPO_5_-peptide resulted in a recognition pattern that is unique for the MPO-specific peptide.

## Conclusion

In conclusion, as shown for the MPO-specific TCR, combination of the immunopeptidomic identification of leukaemia-derived HLA ligands with the isolation of TCR in the single HLA-mismatched setting is suitable to generate highly specific, leukaemia-reactive TCR-transgenic T cells. Further TCR are isolated and characterized at the moment to allow therapeutic application for a broad patient population.

